# Prediction of Damage Accumulation Effect of Wood Structural Members under Long-Term Service: A Machine Learning Approach

**DOI:** 10.3390/ma12081243

**Published:** 2019-04-16

**Authors:** Zheng Li, Duo Tao, Mengwei Li, Zhan Shu, Songshi Jing, Minjuan He, Peng Qi

**Affiliations:** 1 Department of Structural Engineering, Tongji University, Shanghai 200092, China; zhengli@tongji.edu.cn (Z.L.); timbertaoduo@163.com (D.T.); shuzhan@tongji.edu.cn (Z.S.); hemj@tongji.edu.cn (M.H.); 2 Key Laboratory of Performance Evolution and Control for Engineering Structures of Ministry of Education, Tongji University, Shanghai 200092, China; 3 Beijing Deep Singularity Technology Co., Ltd., Beijing 100086, China; limengwei@deepsingularity.com; 4 Department of Control Science & Engineering, Tongji University, Shanghai 201804, China; Jingsongshi1995@gmail.com

**Keywords:** timber structures, damage accumulation model, machine learning, multi-objective optimization, long-term experiment

## Abstract

It is well known that wood structural members can stand a relatively heavy load in the short term but will gradually get weaker if the load is applied for a longer period. This phenomenon is caused by the damage accumulation effect in wood and should be appropriately considered during the design of timber structures. Although various formulation methods (also known as classical models) have been proposed to evaluate the damage accumulation effect in wood, the calibration of model parameters is very time-consuming. Our work proposes a novel method to deal with the damage accumulation effect in wood that involves the application of machine learning algorithms. The proposed algorithm considers a multi-objective optimization process with a combination of goodness-of-fit and complexity. Long-term experimental data of typical wood species are used for developing the machine learning based damage accumulation model. Compared with existing pre-formulated models, our model managed to reduce the complexity of the model structure and give sufficiently accurate and unbiased predictions. This study aims to provide a novel tool for evaluating the damage accumulation in wood structural members, and the proposed model can further support the life-cycle performance assessment of timber structures under long-term service scenarios.

## 1. Introduction

When subjected to long-term loading at high-stress ratios, many materials show a strength reducing effect. Wood is also inevitably influenced by this strength reducing effect during long-term service. This phenomenon is caused by the damage accumulation of wood that is related to the development of internal defects of the material (e.g., initial cracks [1,2], minor splitting [3], etc.). Shu et al. [4] has proposed the analytical approach of the short-term performance of wood members with those internal defects. However, to avoid unwanted structure failure and the consequential economic losses, the damage accumulation should be appropriately considered during the design of timber structures, thus an accurate model is required to make predictions of the strength reducing effect of wood [5]. A few models have been proposed to represent the strength reducing effect of wood, such as Gerhard’s model [6], Barrett and Foschi’s model [7], and Foschi and Yao’s model [8]. However, all the existing models are pre-formulated and based on the empirical generalizations and simplifications, where the unknown coefficients are obtained by a regression process. Taking the Foschi and Yao’s Model (Canadian model) for example, the damage model contains four independent parameters, which are actually equivalent to eight parameters considering the mean value and the standard deviation of each parameter. All the parameters including the model parameters and the short-term performance parameters of wood are modelled as independent lognormal variables, and a non-linear minimization procedure related to the quasi-newton method or genetic algorithm is used to determine the distribution characteristics for each parameter. In order to prevent the interference of the local optimal value, it is often ensured that the global optimal solution is obtained by the combination of continuous optimization of the algorithm and comparison of various algorithms. However, such a process is always time-consuming and difficult to calibrate. This indicates that the damage accumulation of wood has a strong non-linear characteristic and can hardly be interpreted based on existing physical theories or knowledge. Although relatively effective approximations have been made by the classic models, all of them have common problems, such as the model coefficients are too complicated to be interpreted by a mathematical formula, and a fixed expression of formula may never be able to take all those factors into consideration (e.g., wood species, loading history, service conditions, etc.). The limitations of classic models are mainly reflected in the different model parameters for different wood species, which means that long-term experiments on each wood species would be conducted to investigate the long-term performances of different wood species. In addition, if there is no actual wood species corresponding to the parameters to be studied, such as the value and the coefficient of variation of the short-term strength, then it is impossible to determine the parameters of the classic model itself, which would seriously affect the development of subsequent research. Even when the long-term performance of only one wood species is investigated, it is difficult to balance the calculation efficiency and calculation accuracy by the classic mathematical models. Fortunately, with the blooming computer science in the past decade, a more sophisticated model can be generated with a large number of experimental data through the machine learning technique.

Artificial neural network (ANN) is an efficient tool in the realm of machine learning. The reason we seek to employ deep neural network in the prediction of wood damage is that it possesses two essential features that may help us to solve the confronting problems. One is the universal function approximation (UFA) [9]. Theoretically, a forward-single-hidden-layer structure containing a finite number of neurons can approximate any continuous functions, thus any physical processes can be represented by a certain type of network structure. The other feature is the way how neural networks are trained and the way they act. This allows to discover the unknown pattern by directly feeding inputs and labels to the networks, thus saving much laborious work to manually define these features. After the training process, the expected pattern will be grasped and the network should be readily generalized to other similar cases. Such acting manner provides us with a convenient and effective way of adding new input factors into consideration without much work compared with the conventional formulation method (i.e., classical models). As a consequence, the machine learning method by ANN can be used to find the relationship between influencing factors and results for a certain number of experimental data without worrying about the inherent mechanism.

In fact, machine learning has shown its power in many research fields. Except for the most commonly discussed topics on computer vision [10] and speech recognition [11], machine learning has been used in solving complex civil engineering problems. Lu et al. [12] used a double parallel model to forecast underground buried pipeline damage in MATLAB. Lautour and Omenzetter [13] presented a general method to predict seismic-induced damage via ANN describing both structure and ground motion with classical finite element model as comparisons. Machine learning method was used for the prediction of concrete creep in long-term service [14] and for durability and service life assessment of reinforced concrete structures [15]. More recently, machine learning was also introduced into building energy modeling [16] and safety assessment during constructions [17,18,19]. 

Up to the present, there is no relevant research on wood damage prediction using machine learning method. This research seeks to train a neural network model that takes material property, loading quantity, duration effect as well as temporal creep factors as input variables, expecting a damage accumulation and probability forecast as output for Hemlock lumber. Such end-to-end training procedure can give damage rate prediction and requires neither prior knowledge nor empirical formulation used in the classical models. It should be noted that the proposed machine learning model in this paper focuses on the damage accumulation of wood and final failure probability of the wood structural members, while environmental factors like temperature and humidity may be added into the model in future research.

## 2. Methodology

### 2.1. Background

The fundamental concept of ANN is based on the understanding of the way how biological neurons act and operate. Tens of thousands of cells are connected, working concurrently or sequentially through a series of superposition and activation to transform initial simple signals to a complex pattern. Such a unique mechanism unleashes its superiority of self-learning, mapping, and nonlinear functional approximations. This mechanism can capture complex interactions among input/output variables in the system without any prior knowledge about the nature of these interactions, which makes ANN exceptionally a good tool for regression, especially when used for pattern recognition and function estimation.

### 2.2. Algorithms

Steps of training a neural network model can be broken down into the following steps: (1) weights initialization, (2) forward propagation, (3) loss computation, and (4) back propagation algorithm that calibrates the weights. This forward-backward process will be repeated until convergence or the maximum number of iterations is reached.

To start with, Gaussian distribution *N* (0, 0.0001) was used to initialize the network. The initialization scheme gave the weights with a slightly different value from zero that can substantially fasten convergence, as indicated by a former study by Glorot et al. [20].

**Figure 1:**
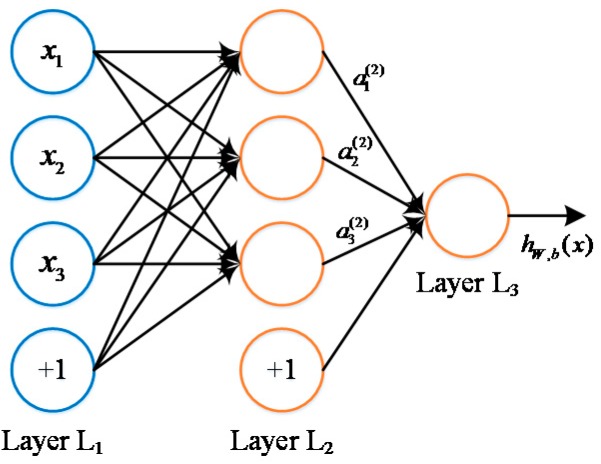
Illustration of a fully connected network.

Forward propagation is one of the key steps in the neural network where simple signals are superposed and activated. As shown in Figure 1, neurons are hooked together forming a fully connected network. Neurons labeled “+1” is called bias units. As can be seen in this figure, layers L1, L2 and L3 denote the input layer, hidden layer and output layer, respectively. Moreover, *x_i_* denotes the *i*th element in an input vector, $a_{i}^{\left(l\right)}$ is the activation on neuron *i* of the layer *l*, $h_{w,b}(x)$ is the final output of the network given an input vector *x* with the weight matrix of *W* and the bias vector of *b*. Each neuron can be regarded as a computational unit that takes in the input vector *x*, performs the weighted sum of inputs, and then activates the weighted sum.

Specifically, for a single unit structure, the total weighted sum of inputs to neuron *i* in layer *l* + 1 ($Z_{i}^{\left(l+1\right)}$) can be expressed as:

(1) $$Z_{i}^{\left(l+1\right)}=\sum_{j=1}^{n}W_{ij}^{\left(l\right)}x_{j}+b_{i}^{\left(l\right)}$$

where $W_{ij}^{\left(l\right)}$ denotes the weight between input *j* in layer *l* and neuron *i* in layer *l* + 1; *x_j_* denotes the *j*th element in an input vector with *n* elements; $b_{i}^{\left(l\right)}$ denotes the bias in layer *l*.

As for a supervised problem with a group labeled data (*x*^(*i*)^, *y*^(*i*)^), when *h_w,b_*(*x*) denotes the non-linear hypotheses given by neural networks under circumstances of parameter (*W*, *b*), and function *f* performs a specific element-wise activation that yields activated vector *a*, the compact notation of forward propagation depicting all neurons at once using matrix operations can be written as:

(2) $$z^{\left(2\right)}=W^{\left(1\right)}x+b^{\left(1\right)}\;a^{\left(2\right)}=f\left(z^{\left(2\right)}\right)\;z^{\left(3\right)}=W^{\left(2\right)}a^{\left(2\right)}+b^{\left(2\right)}\;h_{w,b}(x)=a^{\left(3\right)}=f\left(z^{\left(3\right)}\right)$$

Then the loss *J* can be calculated as:

(3) $$J=\sum_{1}^{N}{\left(Y-h_{w,b}(x)\right)}^{2}/N$$

where *y*^(*i*)^ denotes the ground truth label for the *i*th sample, and *N* is the number of samples. The loss function is given by the Mean-Square-Error (MSE), and in actual use of back propagation, an additional 1/2 is multiplied upon this form to take derivative more easily. Gradients are computed by back propagation method, and the minibatch stochastic gradient descent (SGD) with momentum is implemented to calibrate weights over epochs. The SGD algorithm can be described as:

(4) $$w_{t}=w_{t-1}-\eta \times \nabla_{w}J\left(w;x^{\left(i;i+n\right)};y^{\left(i;i+n\right)}\right)\;v_{t}=\gamma \times v_{t-1}+\eta \nabla_{w}J\left(w\right)\;w_{t}=w_{t-1}-v_{t}$$

where *η* and *γ* stand for the learning rate and the ratio of momentum, respectively. Specifically, these two parameters are set as 0.01 and 0.5 when the weight decay parameter is taken as 1 × 10^−5^ in this study. *w_t_*_−1_ and *w_t_* are the weight for the previous and current time steps, respectively. This algorithm keeps working over iterations of training epochs until convergence or the maximum iteration limit is reached.

## 3. Machine Learning Based Damage Accumulation Model

Wood is stronger under loads of short-term duration and becomes weaker if it is subjected to a long-term, sustained load. This phenomenon results from the time-dependent increase of strain called duration of load (DOL). Since the strength properties of wood are significantly influenced by the DOL effect, design codes for timber structures, such as the Canadian code CSA O86 [21] and the Chinese code GB 50005 [22], specify DOL strength reduction factors during the design of timber structures. It should be noted that DOL effect is an inherent material characteristic of wood instead of a biological process over time. For the long-term experiment under constant loads with a sample size over 300, the primary relationship between the time to failure and the stress ratio (i.e., the ratio of the applied stress over the short-term strength of wood) can be revealed. A damage accumulation model is in need to quantify the DOL effect and to estimate the residual strength of wood under different loading conditions. When calculating the damage state of wood for any loading histories, an approximate load model is adopted, which subdivides the load history into several time intervals with constant applied stress levels. However, complex physical mechanisms and possible chemical interactions have made it difficult to accurately predict the DOL effect using traditional engineering formulas. To address these challenges, a machine learning method is applied to develop a practical nonlinear model for predicting the time-dependent DOL effect of wood in this study.

### 3.1. Long-Term Experimental Data

**Figure 2:**
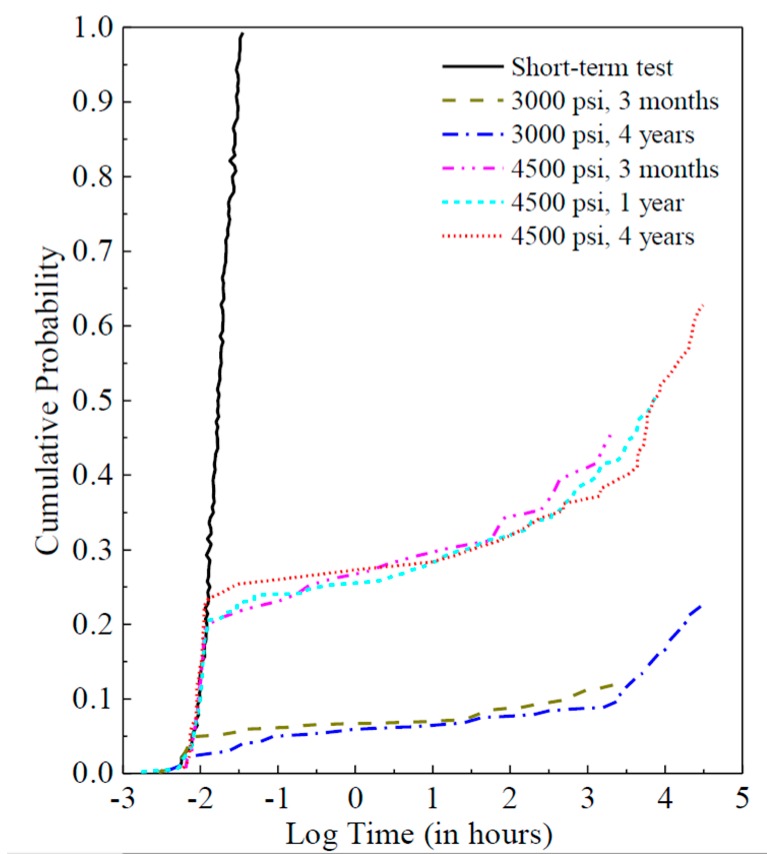
Short-term and long-term experimental results.

The database for this study consisted of five groups of long-term experimental data. The long-term experiments were conducted at FPInnovations Canada, which is a non-profit member organization that carries out scientific research and technology transfer for the Canadian forest industry. The time to failure obtained for both constant stress levels and the controlled short-term tests were ranked, and the corresponding cumulative probability distributions are plotted as shown in Figure 2. Two stress levels were chosen as the long-term constant loading levels, which is 4500 psi (31 MPa) and 3000 psi (21 MPa) corresponding to the 20th-percentile and 5th-percentile of the short-term strength distribution of the lumber, respectively. At the 4500 psi level, for example, approximately 20% of the specimens failed during the loading process, and the cumulative distribution matches quite well with that from the controlled short-term tests. Furthermore, this figure shows that a significant upward departure from the initial trend occurs from the third month with the applied constant stress levels, and such phenomenon is possibly due to the positive-skewed distributions of the influence factors for the damage accumulation model.

**Figure 3:**
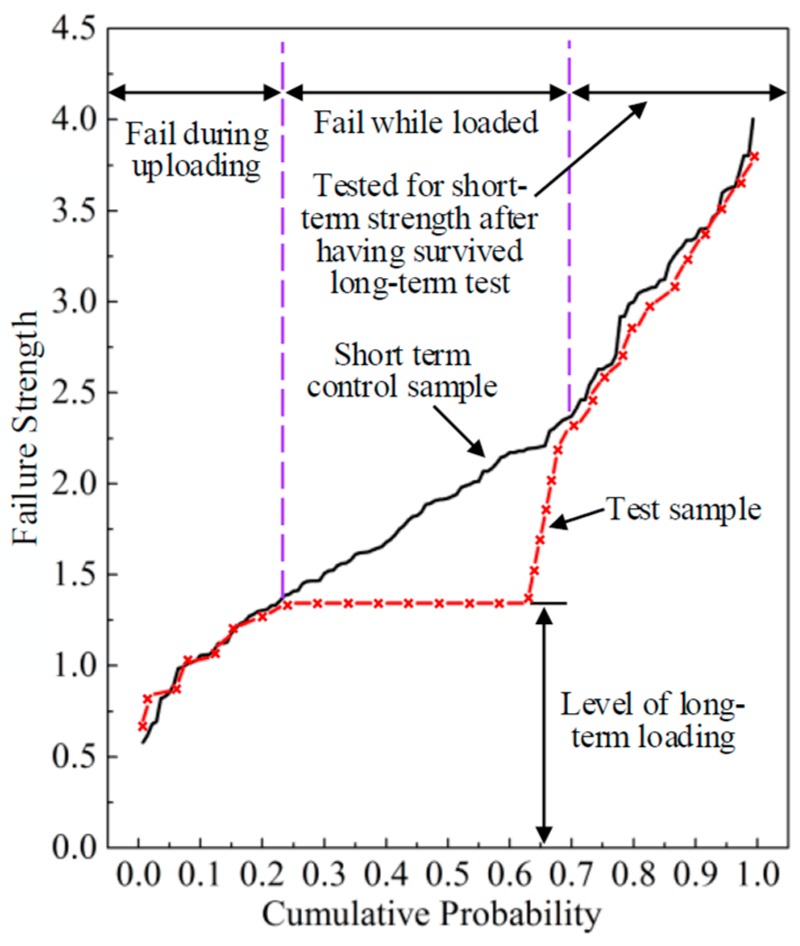
The process of assessing strength reduction.

Although it would be ideal to conduct the experiments as long as possible, most experimental groups were unloaded after three years due to a realistic compromise. The surviving specimens were tested for failure under ramp loading to assess the reduction in short-term strength induced by the period of constant loading. This process is illustrated in Figure 3 where the strength of the control curve is shown versus cumulative probability with normalized rank. The short-term test curve is shown as a solid line while the dotted red line represents the results from a long-term test with a constant load level. It can be observed that the residual strength of the specimens reduced due to the DOL effect. For the survived pieces that subsequently failed in a short term test, the weakest pieces in that group did not match their counterparts on the control curve but that involved only three to five pieces. After that, the residual strength of long-term specimens would match up with its counterpart in the control sample.

The whole database is split into a training dataset and a testing dataset for training and evaluating the network with a ratio of 0.8 to 0.2. The training dataset is randomly shuffled to break down possible sequential dependencies. In general, all inputs need to be dimensionless and standardized to analogous distribution to reduce the difficulty of training. A Z-score function that achieves a deviation of 1 and a mean of 0 can satisfy these requirements and was used in this study, and Tanh activation functions [23] were employed to all hidden layers within the network.

### 3.2. Model Implementation

**Figure 4:**
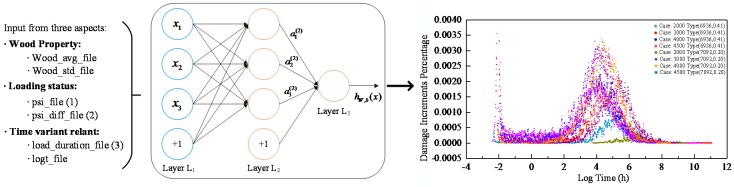
Diagrammatic sketch of input and output on the damage process prediction network model.

The input variables used in the machine learning method were selected according to the selection of parameters in the model proposed by Foschi et al. [8]. The proposed damage accumulation model considers the effect of the diversified wood species that are also treated as network input. After investigating into the inputs form and the condition of long-term experimental data, the selected input variables are as follows: distribution of short-term strength (i.e., wood property that consists of mean value and standard deviation), current applied stress, the variation trend of applied stress, logged load duration, and time since initial loading (i.e., logged hour). All input series and output labels were normalized properly as aforementioned. The structure of the model and an illustration of load-relevant inputs (1, 2, 3) and output are shown in Figure 4. The left column shows the input concerns, including wood property, loading status and time-relevant variants. When all these data are fed into our neural network model, it is expected that the proposed model will be able to generate the increment of damage percentage as output signals through the fully connected network.

One possible understanding of the parameter calibration process in our model is that the model tries to reveal the essence behind the random phenomenon of creep in wood. Such training process of parameter calibrating actually establishes a potentially deterministic relationship between a given input and the target, which would reflect how variables (e.g., damage accumulation) are interdependent (e.g., material properties, load duration, etc.) and attribute it to a causal relationship.

It is commonly acknowledged after 2015’s ImageNet competition that a deeper structure will have better performance under mild assumption that the training data is of good quality (i.e., the data should be sufficient and well distributed). In this study, the network consisted of 10 hidden layers except for the essential input layer and the output layer, and each layer in the network was assigned with an adjustable scale factor (namely the number of neurons) specifying layer width for later evaluation. One hidden layer in the middle was set to have a scale factor twice the value of other scale factors. The hidden layers were activated by TanH, and the output layer was activated by SoftPlus [13] considering label value and the damage process value should be in the range of 0 to 1. Such nonlinear activation functions allowed the networks to compute nontrivial problems using only a small number of nodes. Dropout was exploited with a random dropout rate of *p* = 50%. This was enlightened by the design of networks as presented in Reference [10]. Dropout is a random process of disabling neurons in a layer with a chance of *p*, so the structure relies less on the output of the nodes in the previous layer. This is a well-known method for regularization to reduce over-fitting. However, dropout was not used to test time prediction in this study as they will undermine the consistency over runs.

### 3.3. Optimum Model Selection

The optimum model is expected to have: (1) good performance and (2) efficient model structure with proper parameters. Therefore, our final machine learning based damage accumulation model (i.e., M-D model) should be able to deal with the complexity of training parameters and to provide accurate predictions. The performance of the proposed method was evaluated via the prediction accuracy using the coefficient of determination (*R*). The model complexity was evaluated via the number of layers or nodes/neurons (denoted as scaling factor in this study). Meanwhile, for the model-selection referred to hyper-parameter tuning, cross-validation was taken to decide the best training parameters such as minibatch size and learning rate for momentum SGD method. 

**Figure 5:**
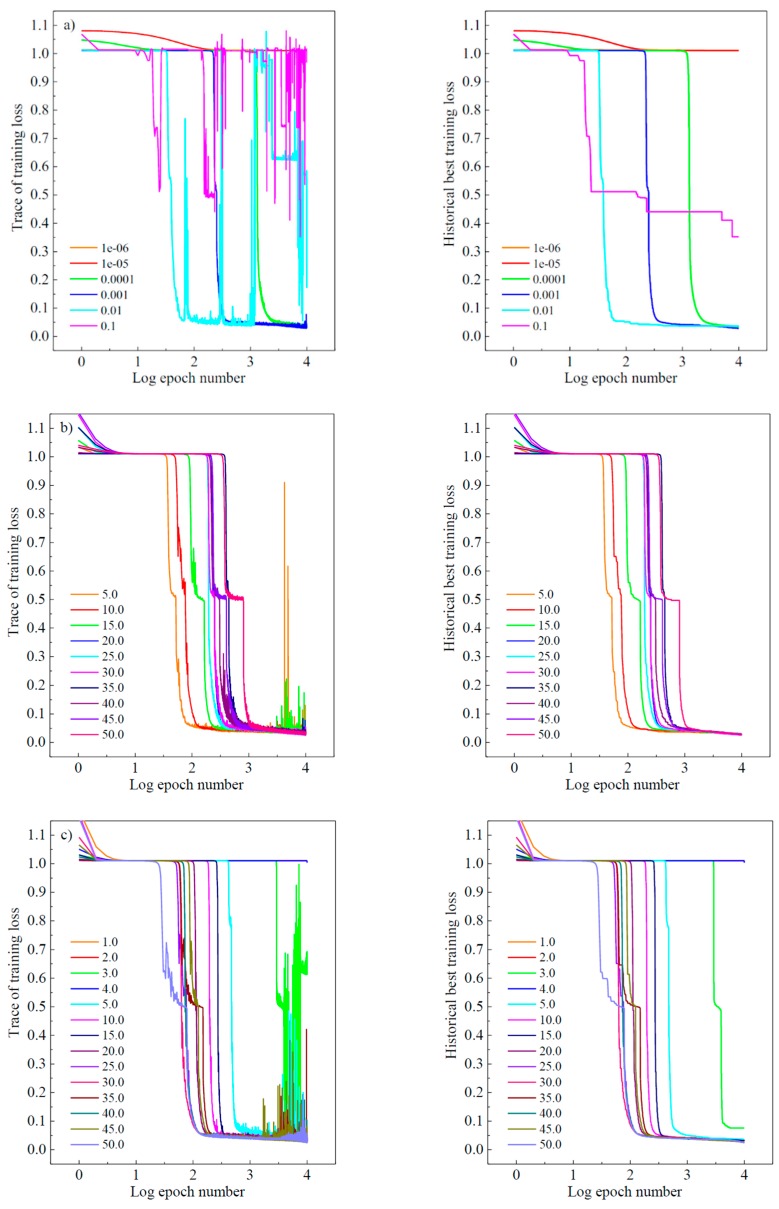
Model-selection: (**a**) algorithm learning rate; (**b**) training data minibatch size; (**c**) model structure scale factor.

It should be noted that all the training parameters were investigated independently during the analytical process. Figure 5 shows the training process during experiments of cross-validation. The trace of training historical loss is recorded on the left and the current best is plotted on the right. The “X-axis” denotes the logged number of the epoch (i.e., each epoch iterates over the whole dataset). During the model selection experiments, the maximum epoch number was set to 10,000. In Figure 5, subplot a) in an upper row shows the training process performance when evaluating the variation of learning rate effect of the momentum SGD algorithm, whilst both the fixed minibatch size and structure scale factor are set to 25. It is noted that when the learning rate is set to be lower than 1 × 10^−^^4^, the optimization speed is relatively low as indicated by the orange and the red line. By setting the learning rate higher than 1 × 10^−2^, it may result in a strong oscillation of the learning curve as indicated by the blue line. Therefore, the learning rate of 1 × 10^−3^ was a reasonable choice towards the best model training result in our specific case.

Similarly, the minibatch and structure scale factor have also been studied, as shown in Figure 5. From subplot b), it is noted that the loss drops faster for a smaller size minibatch. However, the minibatch with the size from 5 to 15 shows a bumpier result in the cost function. In terms of the maximum number of epochs, the training process oscillates with large noise and even fails to converge. The reason regarding the noise is that there were some hard samples which caused gradient oscillations to the cost function. We can see that the larger batch size is, the more it will behave like the full-batch method with a smoother learning curve. Yet learning with a bigger batch is slower and may be quite inefficient when dealing with big training data. Another important issue to be noticed is that there’s an obvious local minimum region in the learning curve. When the loss decreases to about 0.5, a plateau prevents the demanding model to find a deeper relation to describe the material damage. Fortunately, the yellow line with a minibatch size of 25 seems to have a great potential of skipping such a local minimum. Therefore, compromising over training stability, efficiency and effectiveness, the minibatch size of 25 is a proper choice in this study.

The network scale factor was evaluated to decide the network size. With experience within structural engineering, a model giving the coefficient of determination *R* greater than 0.95 will satisfy the lowest requirement for the DOL effect prediction. We gradually increased the number of nodes in each layer until the model behavior met such requirement. This is a reverse-pruning method with the pruning techniques to trim the network size. It was operated by removing nodes from the network during the training by identifying and eliminating unnecessary/redundant nodes that would not noticeably affect the performance of the network. Figure 5c demonstrates that the ability of network is increased when the network size (scale factor) with the scale factor larger than 5, and the model performance achieves a threshold of 0.95. The growing structure of network increases the computational spending of the training process; thus, the size of the net is expected to be as simple as possible in terms of efficiency. In the case where the scale factor is 25, the network can avoid trapping into a local minimum. Meanwhile, the curve shows an expected downward trend and yields an error loss of less than 0.02.

### 3.4. Cross-Validation of the Model

In order to understand the stability of the model, explore the generalization ability of the model and evaluate the accuracy of the model prediction in practice, the cross-validation method was used to verify the model performance and to serve as the basis for the final model selection. In consideration of the small data set, 50 times of random selection were adopted to examine whether the model correctly learned most of the patterns from the data and was not overly disturbed by noise in the data. The results showed that the deviation and variance of the model were both kept at a lower value.

**Figure 6:**
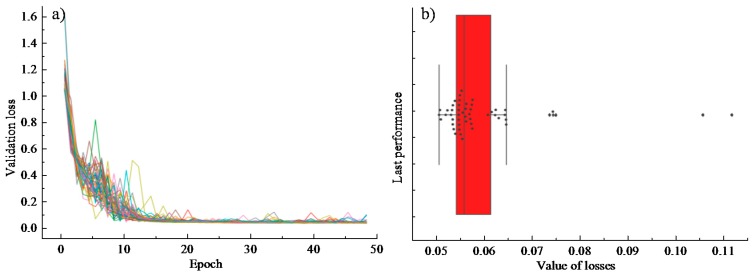
Cross-validation analysis: (**a**) validation loss over 50 times of training; (**b**) model performance at the last cross-validation epoch.

Fifty groups of partition experiments were conducted to access the comprehensive performance of different training processes on different data sets through a certain number of iterations of training. The analysis results are shown in Figure 6. It is noted from Figure 6a that due to the different division of the training set and the test set, the loss value of the objective function shows some differences in the first 15 iterations of training. However, the loss function gradually gets converged as the model training continues. Specifically, the error prediction loss of each group of trainings on the test set is shown in Figure 6b (each point in the box diagram represents an experiment): the mean loss is 0.05923, the standard deviation is 0.01166, and the variance is 0.00014. It is believed that the final effects were acceptable with satisfactory performance of the parametric training. Moreover, the validation results showed that the verification set and the training set of the data satisfy the independent and identical distribution. The final training effect of the model is independent of the number of divisions.

### 3.5. Optimum Model Performance

After investigating several important parameters in model selection, the final M-D model was trained with two phases with parameter settings listed in Table 1. The training process of the final optimum model was done first on Phase 1 that begins from initialization with a relatively bigger learning rate of 1 × 10^−3^ and momentum of 0.5 and then switched to Phase 2 with a smaller learning rate of 1 × 10^−5^ and momentum of 0, respectively.

**Figure 7:**
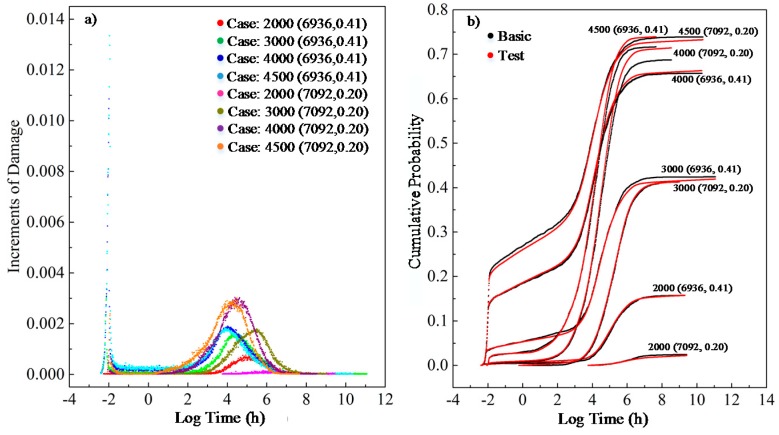
Best M-D model performance on the whole dataset: (**a**) learning output for damage increments; (**b**) cumulative damage result given by M-D model.

The final performance on training and testing data is illustrated in Figure 7. The different case parameters in the figure represent the specimens with different strength characteristics and tested under different loading. For example, the case “2000 (6936, 0.41)” means that the mean value and coefficient of variation of the short-term strength for the specimen is 6936 psi and 0.41, respectively, and the specimen is loaded under 2000 psi. As can be seen in Figure 7a, the colorful scatters represent the predicted damage increments given by our model over time with respect to different cases. The gray and red points in the right plot represent the final damage accumulation procedure over the whole experimental period. At last, the performance of the model, with variant inputs such as wood species and loads, to predict lumber load damage reaches an *R*-value of 99% based on the test dataset.

**Figure 8:**
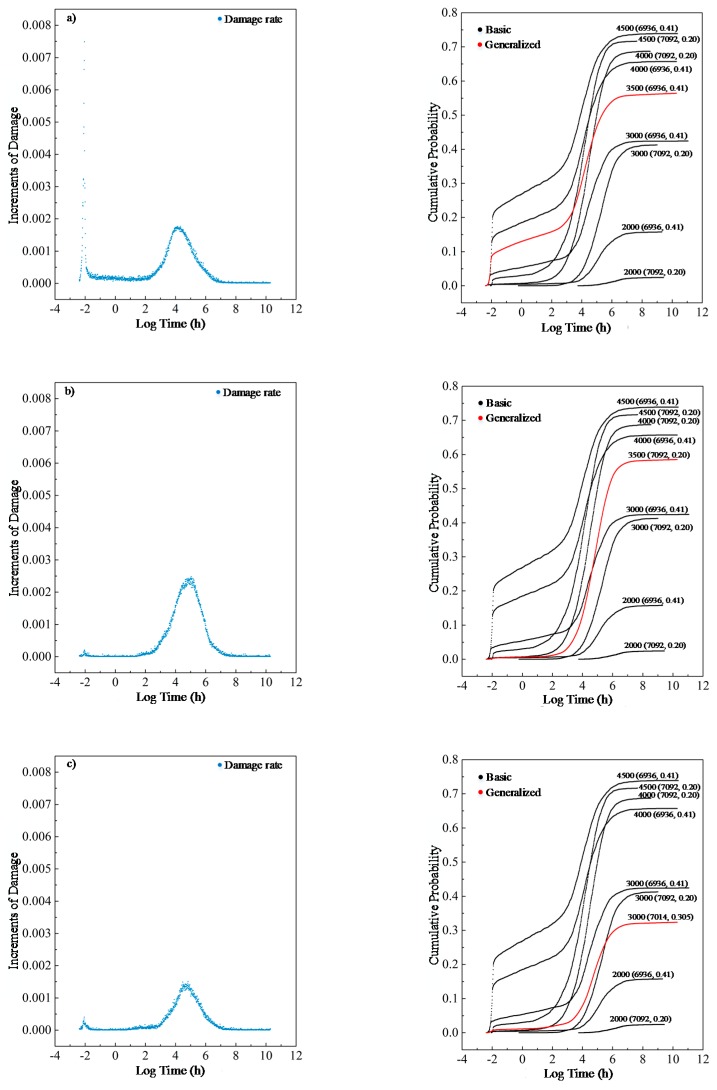
Experiments on model capacity of generalization: (**a**) new load case with a load of 3500 psi and an initial strength of *N* (6936, 0.41); (**b**) new load case with a load of 3500 psi and an initial strength of and *N* (7092, 0.2); and (**c**) new material case with a load of 3000 psi and an initial strength of *N* (7014, 0.305).

Moreover, three experiments were conducted to further demonstrate the model’s ability to generalize other cases as shown in Figure 8.

In Figure 8a,b are new load cases with a load of 3500 psi and an initial strength of *N* (6936, 0.41) and *N* (7092, 0.2), where *N* (*μ*, *σ*^2^) stands for Gaussian Distribution with the mean *μ* and the derivation of *σ*, and c) is a new material case with a load of 3000 psi and an initial strength of *N* (7014, 0.305). The corresponding results are shown in Figure 8 with process damage on the left. All these data are modified from presently available data. As illustrated in Figure 8, for an unprecedented load of 3500 psi, the model performs excellently with both types of lumber. For example, in case of b), the effect of an unprecedented load of 3500 psi on the wood of initial strength *N* (7092, 0.2) is within the range of impact caused by a load of 3000 and 4000 psi. Concurrently, regarding real experiments of psi 3000 and *N* (7092, 0.2) as a reference (as marked), it is found that a decrease in material strength to *N* (7014, 0.305) would deteriorate the load tolerance of wood, which will also be true in the real cases. These facts demonstrate that the M-D model has acquired a few meaningful aspects to predict wood damage behavior.

## 4. Comparisons with Conventional Models

**Figure 9:**
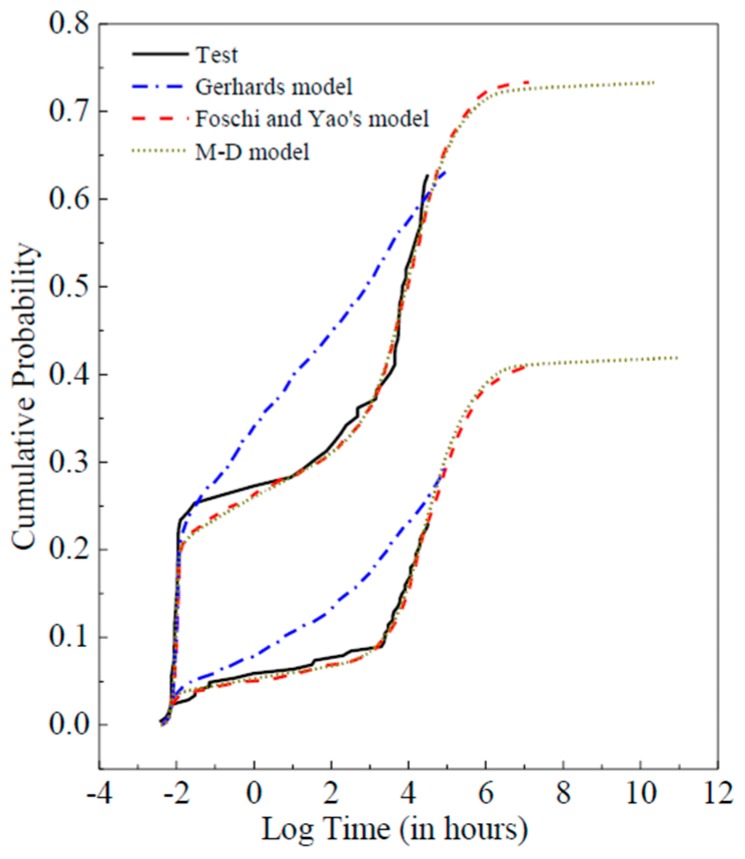
Comparison of different prediction models.

The influencing factors of DOL effect in wood varies and sometimes are highly interactive, which makes it difficult to predict damage accumulation for different wood species with classic engineering models. Meanwhile, many meaningless yet important parameters remain to be calibrated through a whole set of fatigue procedure [16]. Whereas, with the state-of-the-art computational paradigm of the deep learning model, it is not necessary to consider the various source of inputs, complex physical or chemical interaction mechanism when making the lumber damage prediction. Compared with traditional methods, the ANN-based model proposed in this study can tolerate relatively inaccurate, noisy or incomplete data [10]. The proposed model is not susceptible to outliers due to its better filtering capability and better adaptability in most cases. Figure 9 shows the comparisons between the proposed M-D model and the two conventional models (Gerhard’s model and Foschi and Yao’s model). It is noted that Gerhard’s model is not fitted to follow the experimental trends. Nevertheless, based on the proposed model, a rather precise result (*R* > 99%) can be achieved with the above-mentioned superiorities, and it is better or at least equivalent to the results provided by the commonly-used classical models.

Some factors including the environment temperature and humidity and the material density were still not considered in the machine learning model. In general, the material density of wood is considered to be proportional to its short-term strength. Moreover, it should be noted that many of the parameters shown in the formulation of the Foschi and Yao’s model are of no clear physical meaning and just the mathematical parameters introduced to simply fit the complex long-term performance curve. The machine learning model only considers the physical meaning parameters so as to exclude interference from unrelated variables on the model mechanism.

## 5. Conclusions

Neural networks build interactions between input features and automatically generate the best-fit model. With the help of computer science techniques, this study proposed a novel machine learning method for complex civil engineering problem (i.e., time-dependent damage accumulation of wood).

To simultaneously optimize objectives of the best performance and the model complexity, model structure selection and parameter calibration experiments were carried out with consideration on goodness-of-fit. In terms of model design, learning rate and training batch size are important to be set properly so that weights optimization algorithm can overcome difficulties during the training process, and the scale of the model structure may directly limit the capacity of net learning pattern. With the final optimum model setting indicated by model selection experiments, our prediction model achieves *R* score of 0.9938. Moreover, when this M-D prediction model is generalized to unprecedented load or over different wood property, it can still give out some meaningful indicative references on unseen cases. Besides, the proposed method avoids the fatigue process of parameter calibration, and meanwhile, it can take other potentially influential factors into consideration by just enlarging the dimension of input features. Though there are still some drawbacks for such machine learning model, such as the inability to consider all the wood species and any time interval, it is believed that the model has certain application prospects and provides a reference for subsequent research. This is due to that the experimental data used for the model training process has covered a wide range of wood species from the perspective of the mean value and coefficient of variation of the wood strength. In terms of wood structure design, the most potential application of such machine learning model is to obtain the load duration influence coefficient under different load conditions and different live/constant load ratios through reliability analysis. Such coefficient would make a direct influence on the conversion of wood properties from the standard value to the design value under the long-term effect of load, resulting in a more effective and economical design index of wood structures considering the whole life cycle. Moreover, the potential for predicting the long-term damage accumulation is mainly reflected in the ability to further study the damage process within each individual wood member.

This study tries to provide a tool for quantifying the damage accumulation effect of wood structural members and can further support the life-cycle performance assessment of timber structures on a larger scale.

## Figures and Tables

**Table 1:** Configurations for training optimum model.

Phase Label	Algorithm	Learning Rate	Weight Decay	Momentum	Minibatch Size	Scale Factor	Epoch
Phase 1	SGD	0.001	1 × 10^−5^	0.5	25	25	10,000
Phase 2	SGD	0.0001	1 × 10^−5^	0	25	25	20,000
